# Comparative histological analysis of the skin for forensic investigation of some animal species

**DOI:** 10.17179/excli2022-5335

**Published:** 2022-10-26

**Authors:** Elsayed S. I. Mohammed, Fatma A. Madkour, Mohammed Zayed, Rasha Radey, Ahmed Ghallab, Reham Hassan

**Affiliations:** 1 Department of Histology and Cytology, Faculty of Veterinary Medicine, South Valley University, Qena 83523, Egypt; 2 Department Anatomy and Embryology, Faculty of Veterinary Medicine, South Valley University, Qena 83523, Egypt; 3 Department of Surgery, Faculty of Veterinary Medicine, South Valley University, Qena 83523, Egypt; 4 Animal Health Research Institute, Qena Branch, Qena, Egypt; 5 Department of Forensic Medicine and Toxicology, Faculty of Veterinary Medicine, South Valley University, Qena 83523, Egypt

**Keywords:** forensic medicine, skin histology, epidermis, differential histology

## Abstract

Macroscopical and histological analysis of the skin is fundamental in both human and veterinary forensic investigations. However, databases of differential skin histology of various animal species are rare. The aim of the present study is to identify species-specific differential histological features of the skin that could be used in forensic investigations including animal identification. For this purpose, skin specimens were collected from the neck region of various farm animals including buffalo, cow, camel, sheep, goat, dog, and donkey, and were processed for histological analysis. Our comparative analysis revealed specific histological features in the skin that could differentiate between the studied animal species. The epidermis layer of the skin was very thick in buffalo, intermediate in cow, sheep, goat, dog, and donkey, but very thin in camel. The papillomatous epidermis was very frequent in buffalo, but very rare in cow. In the dermis layer of the skin, four structures were located which showed differential features between the studied animal species: *the papillary layer*, which was thick in buffalo, camel, sheep, dog, and donkey but thin in cow and goat. *The sweat glands*, which were few in buffalo, cow, goat, and dog, but numerous and deeply located in the dermis of sheep; they were individually located in all studied animals except in camel and donkey they were arranged in clusters. *The hair follicles* were characteristic for the skin of sheep as they were present at two different levels in the dermis with simple and compound hair follicles. *The sebaceous glands* were large and multi-lobular in buffalo, but small and uni-lobular in cow and camel. The hypodermis layer of the skin was very thick in sheep and goat in contrast to all other analyzed animals. In conclusion, the present study provides comprehensive information on the differential histological features of the skin of seven different domestic animal species that could be used as a key in forensic investigations.

## Introduction

Skin is the largest organ that covers the external surface of the body and forms a barrier between the internal organs and the external environment. Histologically, the skin consists of three main layers; the epidermis, the dermis and the hypodermis (Kanitakis, 2002[20]). The epidermis is composed of keratinized stratified squamous epithelium of multiple layers: basal, spinosum, granulosum, and corneal layer. It is free of blood vessels, and it receives the oxygen supply by diffusion from the dermis. The dermis layer is composed of a papillary layer, which contains reticular fibers and blood capillaries, and a reticular layer, which includes bundles of collagenous fibers; the dermis layer hosts important structures such as the hair follicles, the sweat glands, and the sebaceous glands. The inner most layer of the skin is the hypodermis which consists mainly of fat cells and loose connective tissue (Arda et al., 2014[2]; Prost-Squarcioni, 2006[29]).

Skin inspection is of fundamental importance in forensic investigations, in both antemortem and postmortem examination (Bardale et al., 2012[4]; Reddy and Lowenstein, 2011[30]). This includes: (i)* estimate the time elapsed after death *by analysis of macroscopical and histological postmortem changes of the skin. Body cooling (also called algor mortis) is one of the earliest changes in the skin that occurs within minutes after death (Lee Goff, 2009[24]; Reddy and Lowenstein, 2011[30]). This is followed by hypostasis (also known as postmortem lividity) which is the accumulation of blood in the lower parts of the body; it is evident as early as 30 minutes after death and becomes very obvious within 6 hours (Lee Goff, 2009[24]). The next postmortem change is the rigor mortis or muscle stiffness; it starts relatively later than hypostasis and occurs initially in the face and the neck followed by the other body parts (Lee Goff, 2009[24]; Reddy and Lowenstein, 2011[30]). Body decomposition is a late sign of death which becomes evident within 24 hours and manifests by greenish discoloration of the skin. (ii) In living individuals, macroscopical, histological as well as biochemical analyses of the skin allow to *estimate the wound age* (Kondo, 2007[23]; Oehmichen, 2004[28]; Reddy and Lowenstein, 2011[30]), and the *causal instrument *(Giraudo et al., 2016[16]). (iii) A further key importance of skin examination in forensic investigations is the *identification of a suspected assailant*. A classical approach in this field is to locate and measure imprint marks, and to link assailant to a victim (Reddy and Lowenstein, 2011[30]). In addition, analysis of skin microbiota may allow to identify a suspected assailant; due to the high interindividual variability of skin bacterial communities, analysis of the remaining bacteria on an object could help in forensic identification by matching them with those on the skin of a suspected assailant (Fierer et al., 2010[14]; Neckovic et al., 2020[27]). Although the field of microbiota analysis is advanced in other body organs, e.g., the gut (Schneider et al., 2021[31][32]), skin microbiota and their role in forensic identification is still on its infancy stage. (iv) An additional key role of skin inspection especially in veterinary forensics is the *analysis of wound vitality *(Casse et al., 2016[11]). This is particularly important to avoid meat adulteration by differentiation between antemortem and postmortem slaughtering of animals. The presence of a gap between the wound edges, extensive bleeding, coagulation, inflammation and swelling of the wound edges, as well as elevation of acid and alkaline phosphatases indicate that slaughtering occurred before death and vice versa (Reddy and Lowenstein, 2011[30]). 

Comparative analysis of the histological structure of the skin layers is also of medicolegal importance in animal species identification. However, detailed databases of comparative skin histology in various animal species are rarely found. The aim of the present study is to establish a database comparing skin histology in buffalo, cow, camel, sheep, goat, dog and, donkey to identify species-specific skin features that allow animal identification in forensic investigations. 

## Materials and Methods

### Sample collection

Skin specimens were collected from healthy adult buffaloes (Bubalus bubalis, 2.5-3-years-old), cows (Angus cattle, 2.5-3-years-old), camels (one-hump dromedary camel, 6-7-years-old), small ruminants (Egyptian goat and Rahmani sheep, 1-1.5-years-old), dogs (Egyptian Baladi dog, 9-12-months-old), and donkeys (African domestic donkey, 2-3-years-old) from the animal farm of the Faculty of Veterinary Medicine (South Valley University, Egypt). All specimens were collected from the skin of the neck region. First, the hair was clipped, skin was disinfected, and then a local anesthesia was applied using lidocaine 2 % (Sigma-Aldrich). After desensitization of the skin, a specimen was collected, and the wound was sutured using non-absorbable suture material. The samples were collected from at least three animals of each species, and representative images are shown in the result section. The samples were collected and handled in accordance with the guidelines of the institutional animal ethics committee of South Valley University (Stilt, 2018[34]), application number 47/12.09.2022.

### Sample processing

Immediately after collection, the samples were washed in physiological saline, and were then fixed in 10 % neutral buffered formalin for 24 hours. Subsequently, the samples were dehydrated in an ascending ethanol series, cleared in methyl benzoate, and finally embedded in paraffin wax. Four μm-thick formalin-fixed paraffin-embedded tissue sections were prepared using a microtome (Leica RM2235, Leica Biosystems).

### Histological staining and image acquisition

To compare the histological structure of the skin in various farm animals, standard staining protocols including hematoxylin and eosin (H&E), Periodic acid-Schiff (PAS), Alcian blue, and Crossman's trichrome stain were applied (Bancroft et al., 2013[3]; Campos et al., 2014[10]; Ghallab et al., 2022[15]; Holland et al., 2022[18]). The stained tissue sections were examined using Leitz Dialux 20 microscope, and representative images were acquired using a Canon digital camera (Canon Power shot A95).

## Results

To identify species-specific characteristic features in skin histology, various histological stainings were performed. Generally, the skin of domestic animals consists of three layers: the epidermis, the dermis, and the hypodermis. Our analysis revealed variability in these layers and in the associating substructures according to the animal species.

### Histological features of buffalo skin

**Figure 1:**
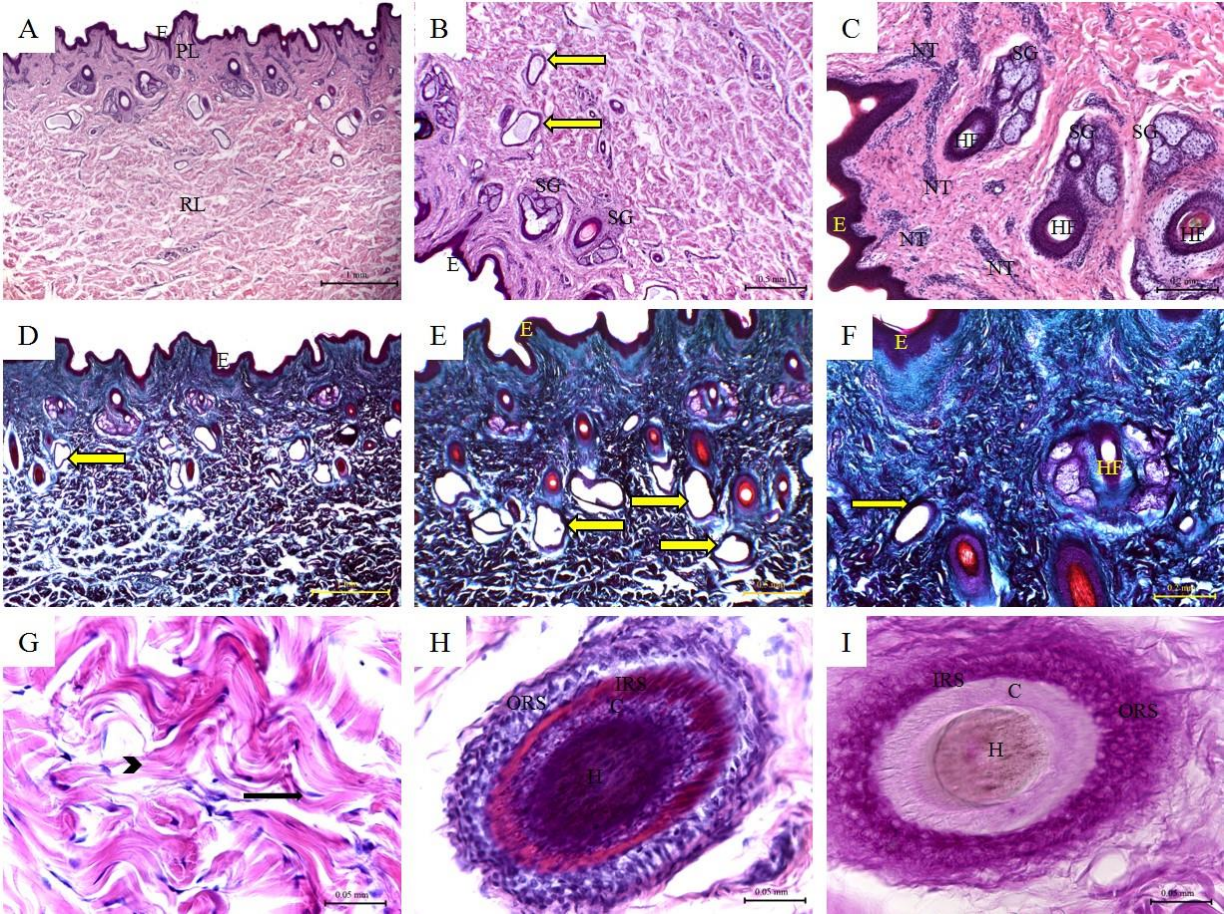
Histological features of buffalo skin. Hematoxylin and eosin staining (A-C; G-H); Crossman's trichrome staining (D-F); and PAS staining (I) of buffalo skin tissue sections. (E) Keratinized stratified squamous epithelium of the epidermis; (PL) Papillary layer of the dermis; (RL) Reticular layer of the dermis; (NT) Nerve tissue; (SG) Sebaceous glands; (Black arrow) fibrocytes; (Arrow heads) collagen bundles of the reticular layer; (Yellow arrow) Sweat glands; (HF) Hair follicles; (H) Hair; (C) Cuticle; (IRS) Inner root sheath; (ORS) Outer root sheath.

In buffalo, the epidermis was lined by keratinized stratified squamous epithelium, with clear stratum germinativum and a horny layer. The epidermis is characterized by the presence of multiple downgrowth between the dermal papillae, which are known as papillomatous epidermis. The upper surface of the dermis formed multiple projections upwards giving the shape of a corrugated layer known as the papillary layer. The papillae were simple and varied in length and shape from long and cylindrical to short and thick. The dermis contained numerous fiber types and few cell types, numerous blood and lymphatic vessels, nerves, arrector pili, sweat glands, sebaceous glands, hair, and hair follicles. The dermis was rich in collagen and contained few elastic and reticular fibers, which often were intertwined with each other. The collagen fibers were arranged in bundles and appeared green using Crossman's trichrome staining. The collagen fiber bundles were thicker in the reticular layer than in the papillary layer. The arrector pili was a thin smooth muscle bundle that was in the papillary layer and extended up to the epidermis. The sebaceous glands were numerous, well developed and distributed around the hair follicles. The hair follicles were composed of the dermal root sheath, outer root sheath and inner root sheath (Figure 1(Fig. 1); Table 1(Tab. 1)).

### Histological features of cow skin

**Figure 2:**
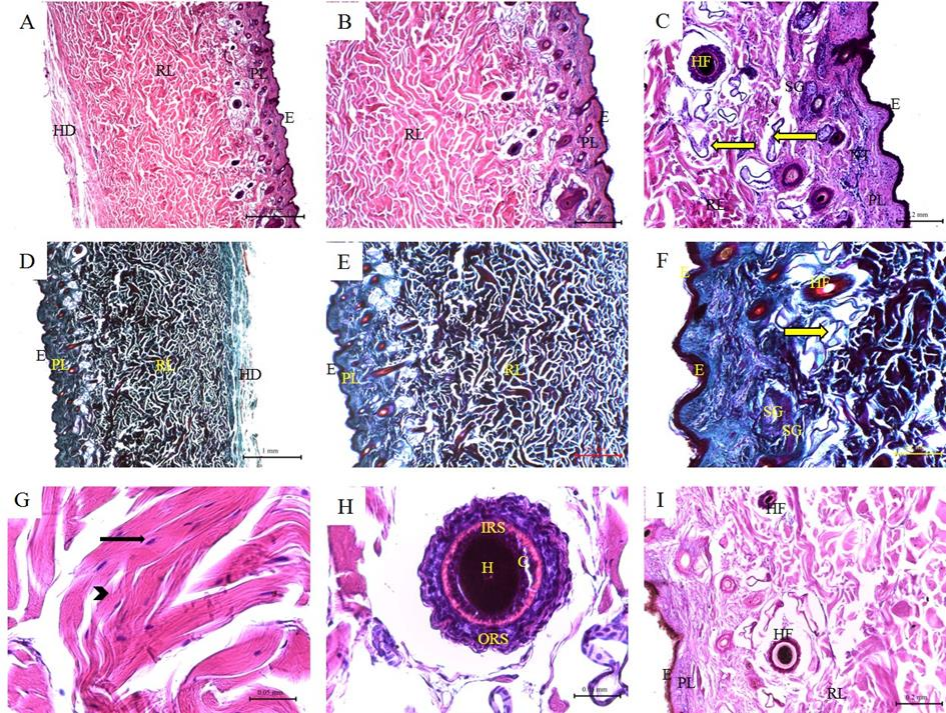
Histological features of cow skin. Hematoxylin and eosin staining (A-C; G-H); Crossman's trichrome staining (D-F); and PAS staining (I) of cow skin tissue sections. *(E) Keratinized stratified squamous epithelium of the epidermis; (PL) Papillary layer of the dermis; (RL) Reticular layer of the dermis; (HD) Hypodermis; (SG) Sebaceous glands; (Black arrow) fibrocytes; (Arrow heads) collagen bundles of the reticular layer; (Yellow arrow) Sweat glands; (HF) Hair follicles; (H) Hair; (C) Cuticle; (IRS) Inner root sheath; (ORS) Outer root sheath. *

The histological structure of the skin of cow showed similar features to that of buffalo with some differences. The epidermis was relatively thinner. The papillomatous epidermis was rarely seen in the epidermis of cow. The papillary layer was thin and contained the skin appendages. The hair follicles were smaller, and each hair follicle was associated with only one or rarely two sebaceous glands. The sweat glands were smaller and more coiled. The collagen bundles were much thicker and densely packed in the reticular layer than in the papillary layer (Figure 2(Fig. 2); Table 1(Tab. 1)).

### Histological features of camel skin

**Figure 3:**
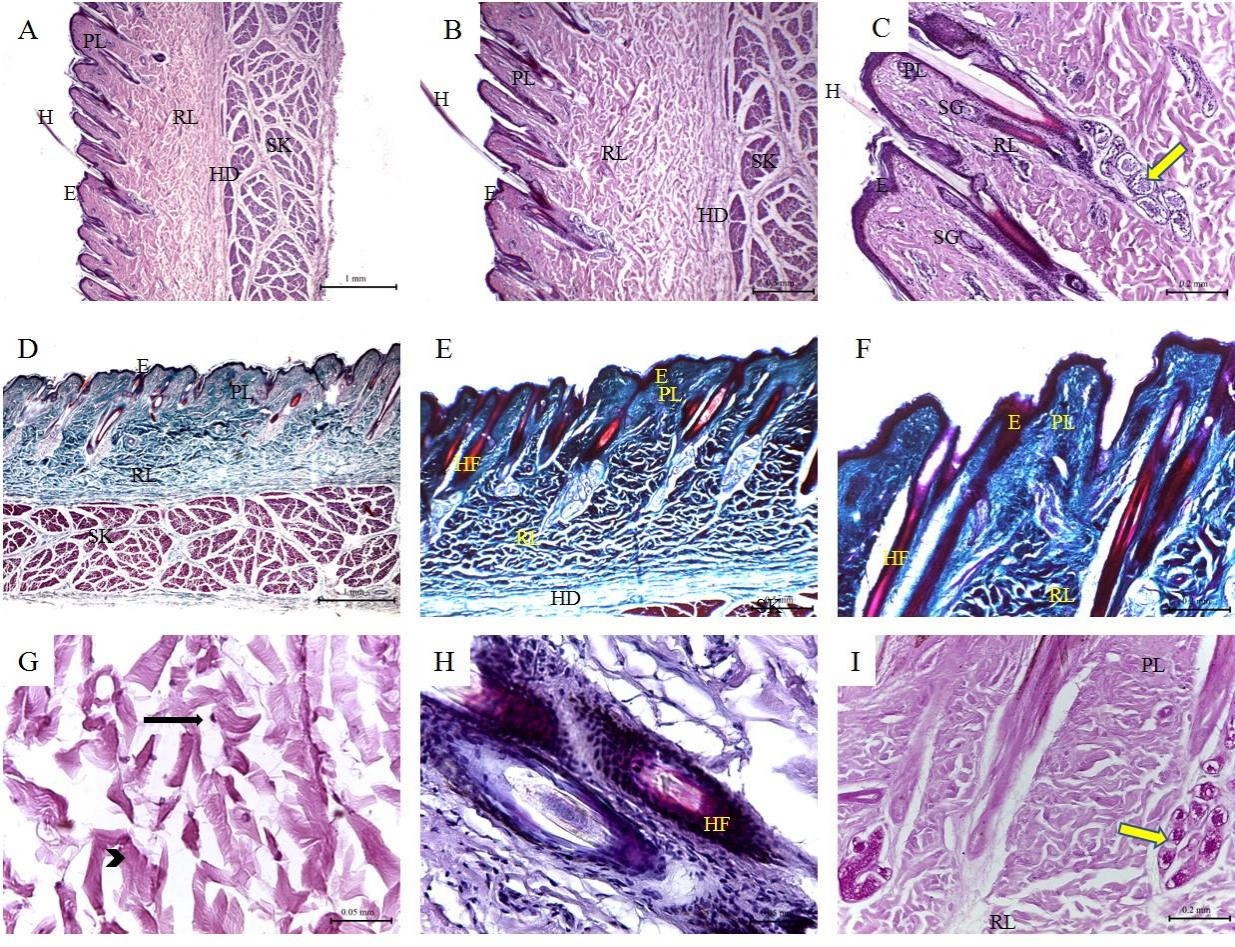
Histological features of camel skin*.* Hematoxylin and eosin staining (A-C; G-H); Crossman's trichrome staining (D-F); and PAS staining (I) of camel skin tissue sections. (E) Keratinized stratified squamous epithelium of the epidermis; (PL) Papillary layer of the dermis; (RL) Reticular layer of the dermis; (SG) Sebaceous glands; (Black arrow) fibrocytes; (Arrow heads) collagen bundles of the reticular layer; (Yellow arrow) Sweat glands; (HF) Hair follicles; (NT) Nervous tissue; (SK) Skeletal muscles.

In camels, the skin epidermis was thin with numerous pigmented cells in the stratum germinativum. The papillary layer formed numerous dermal papillae. The skin appendages in camel were characterized by the presence of numerous sweat glands around the hair follicles. The sebaceous glands were single or paired distributed and were present in attachment with the hair follicles. The reticular layer was formed of collagenous bundles followed by thin hypodermal layer. A skeletal muscle layer was closely attached to the hypodermis in camel (Figure 3(Fig. 3); Table 1(Tab. 1)).

### Histological features of the skin of sheep and goat

**Figure 4:**
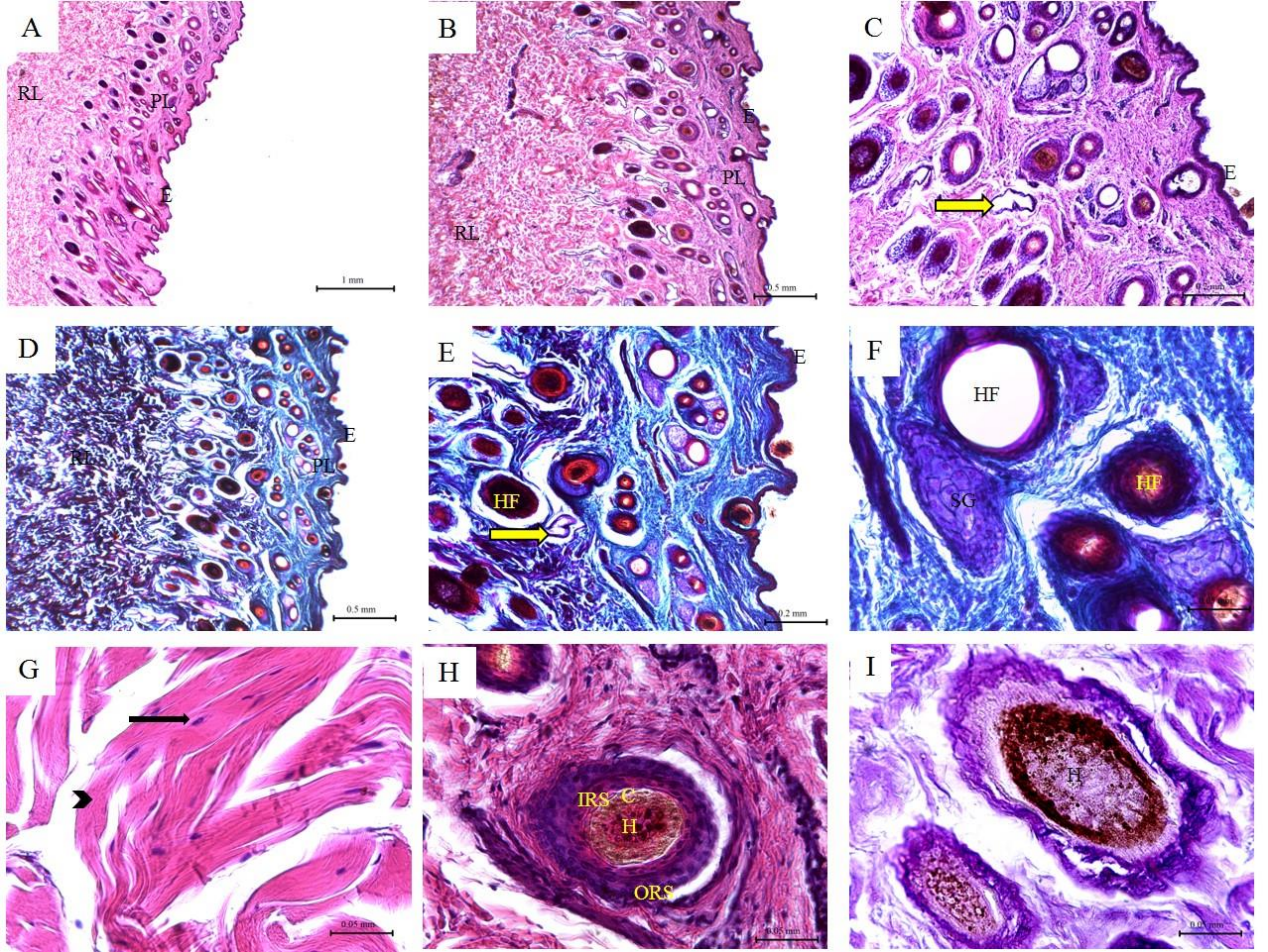
Histological features of sheep skin. Hematoxylin and eosin staining (A-C; G-H); Crossman's trichrome staining (D-F); and PAS staining (I) of sheep skin tissue sections. (E) Keratinized stratified squamous epithelium of the epidermis; (PL) Papillary layer of the dermis; (RL) Reticular layer of the dermis; (NT) Nervous tissue; (SG) Sebaceous glands; (Black arrow) fibrocytes; (Arrow heads) collagen bundles of the reticular layer; (Yellow arrow) Sweat glands; (HF) Hair follicles; (H) Hair; (C) Cuticle; (IRS) Inner root sheath; (ORS) Outer root sheath.

**Figure 5:**
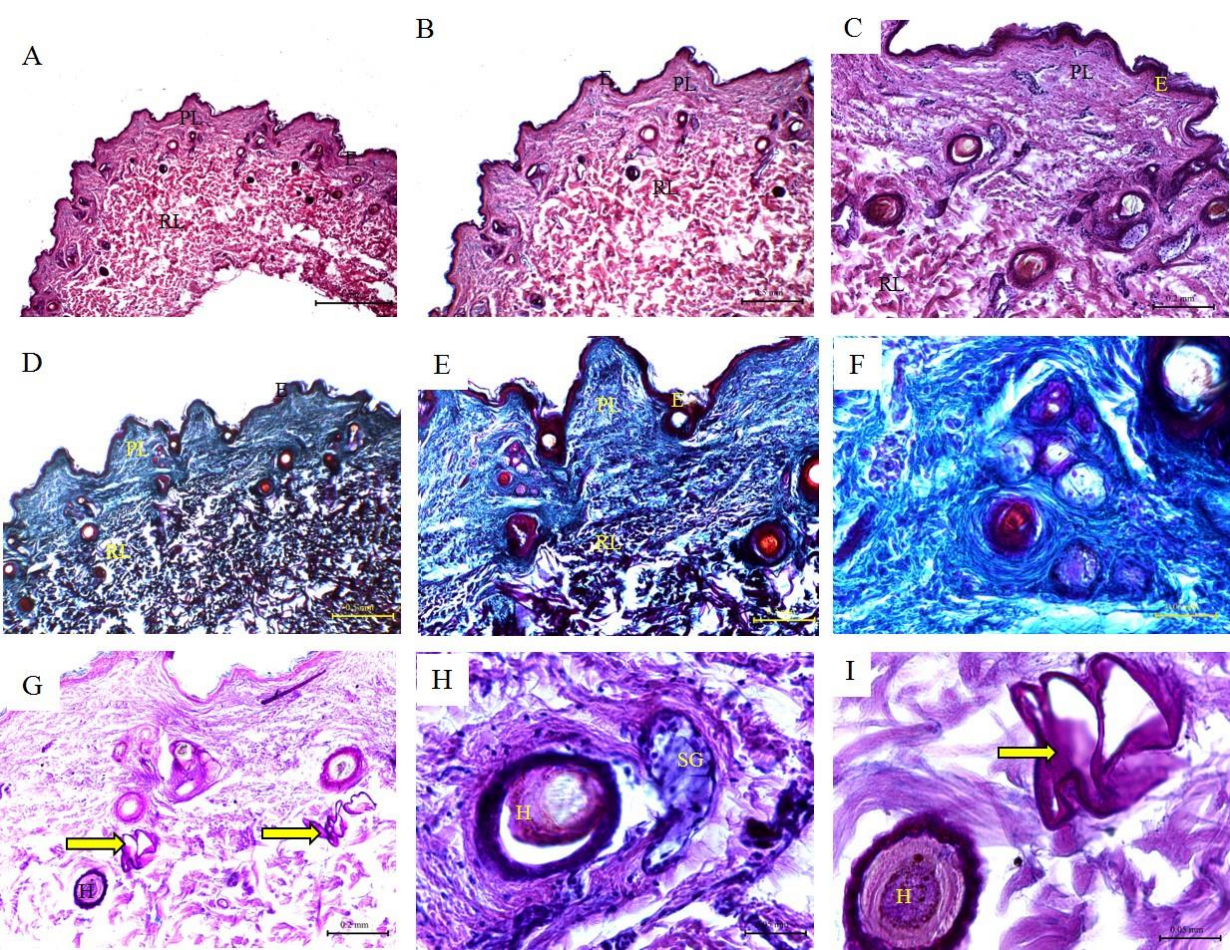
Histological features of goat skin. Hematoxylin and eosin staining (A-C; G-H); Crossman's trichrome staining (D-F); and PAS staining (I) of goat skin tissue sections. *(E) Keratinized stratified squamous epithelium of the epidermis; (PL) Papillary layer of the dermis; (RL) Reticular layer of the dermis; (NT) Nervous tissue; (SG) Sebaceous glands; (yellow arrow) Sweat glands.*

The skin of the sheep consisted of a thin epidermis and a relatively thin dermis with thick hypodermal layer. The reticular layer formed dermal papillae. The sebaceous glands were found in association with the primary hair follicles as single or paired glands. The sweat glands were found in a deeper position in the dermis. The hair follicles were found at two levels at the dermis where simple and compound hair follicles can be observed. The collagen bundles of the reticular layer were thick and similar to that of cows (Figure 4(Fig. 4)). The goat skin showed less dermal papillae, less hair follicles, less sebaceous glands. The sweat glands were few and more coiled (Figure 5(Fig. 5); Table 1(Tab. 1)).

### Histological features of dog skin

**Figure 6:**
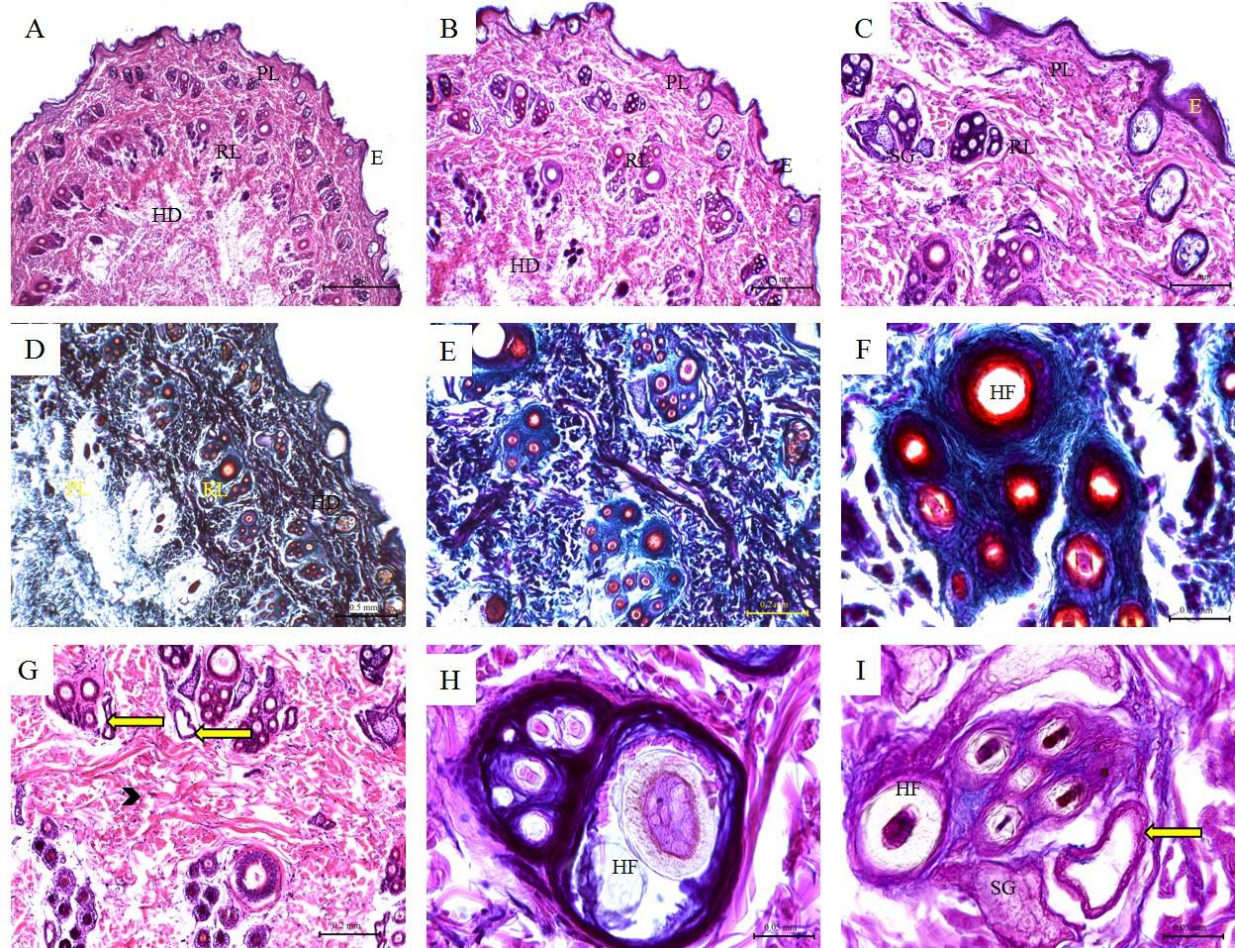
Histological features of dog skin. Hematoxylin and eosin staining (A-C; G-H); Crossman's trichrome staining (D-F); and PAS staining (I) of dog skin tissue sections. E) Keratinized stratified squamous epithelium of the epidermis; (PL) Papillary layer of the dermis; (RL) Reticular layer of the dermis; (SG) Sebaceous glands; (Arrow heads) collagen bundles of the reticular layer; (Yellow arrow) Sweat glands; (HF) Hair follicles.

The skin of dogs showed a thin epidermis with few epidermal papillae. The dermis formed of papillary and reticular layers where numerous compound hairs were found and formed of multiple secondary hairs ranged from 5 to 7 and single primary hairs. A few sweat and sebaceous glands were found in association with the hair follicles (Figure 6(Fig. 6); Table 1(Tab. 1)). 

### Histological features of donkey skin

**Figure 7:**
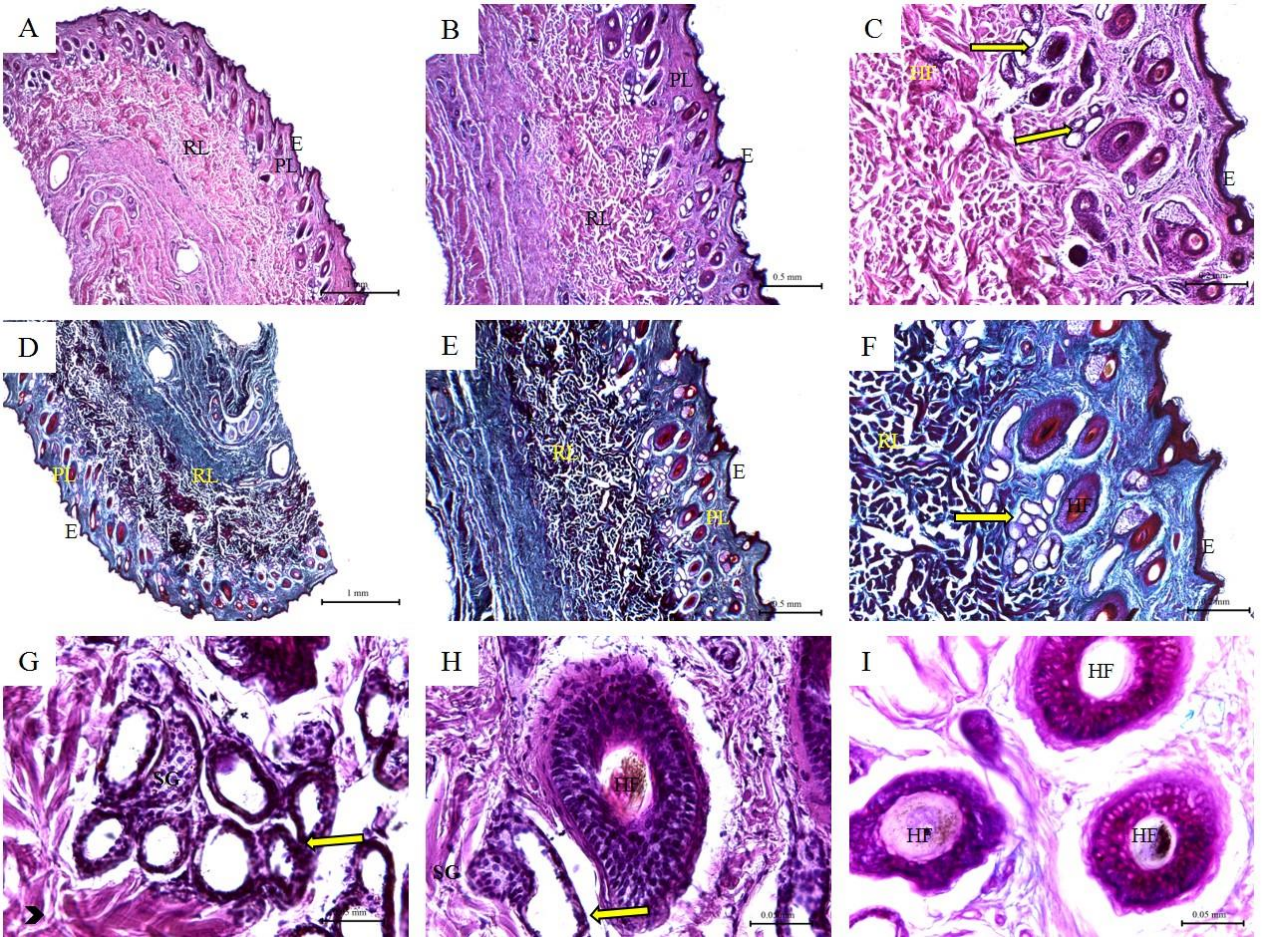
Histological features of donkey skin. Hematoxylin and eosin staining (A-C; G-H); Crossman's trichrome staining (D-F); and PAS staining (I) of donkey skin tissue sections. (E) Keratinized stratified squamous epithelium of the epidermis; (PL) Papillary layer of the dermis; (RL) Reticular layer of the dermis; (SG) Sebaceous glands; (Arrow heads) collagen bundles of the reticular layer; (Yellow arrow) Sweat glands; (HD) hypodermis; (HF) Primary follicle of the compound hair surrounded by secondary follicles.

The epidermis of the skin of donkey was thin with few epidermal apparatuses. The dermis formed of papillary and reticular layers. The skin appendages include few sebaceous glands, clusters of sweat glands and single hair follicles. The collagen bundles of the reticular layer were very thick and contained numerous nerve tissue (Figure 7(Fig. 7); Table 1(Tab. 1)).

## Discussion

Although histological analysis of the skin is common in human forensic investigations, e.g., it helps in estimation of a wound age (Betz, 1994[5]; Casse et al., 2016[11]) and the time elapsed after death (Wei et al., 2020[36]), its application in veterinary forensics is not well developed. The main obstacle is that reference databases of skin histology of various animals are rare. In the present study, we report species-specific differential histological features of the skin layers that could be used in veterinary forensic investigations. Specifically, histological structure of the skin layers and the associated substructures were compared in buffalo, cow, camel, sheep, goat, dog, and donkey. Based on the diameter of the epidermis and the hypodermis, the frequency of the papillomatous epidermis, the diameter of the papillary layer of the dermis as well as the number of the sweat glands species-specific characteristic features were identified (Table 1(Tab. 1)). Histology of the skin of both male and female animals was analyzed, but we did not observe any obvious gender-related differences. Our results agree with previous reports on animal skin histology (Bhat et al., 2014[6]; El-Shafey et al., 2017[13]; Ibrahim and Hussin, 2017[19]; Ludewig et al., 1996[25]; Meyer and Neurand, 1987[26]) but with few exceptions. The study of Ibrahim and Hussin (2017[19]) reported that the sweat glands of buffalo skin are poorly developed. In our analysis, although the sweat glands of buffalo are relatively few in number compared to the other studied animals, they are well-developed. A possible explanation of this discrepancy is that the study of Ibrahim and Hussin (2017[19]) was conducted on the skin specimens collected from slaughtered animals, in contrast to our analysis which was performed in skin samples collected from living animals. Ludewig et al. (1996[25]) reported that the sweat glands are numerous in cow skin. This is in contrast to our study which shows relatively less frequent sweat glands in the skin of cows. Since the skin specimens were collected from different regions, the observed discrepancy could be attributed to the regional variation of the sweat glands distribution in the skin (Debbarma et al. 2018[12]).

Animal identification is of particular importance in forensic investigations. Analysis of animal remains such as hair or bones is commonly applied to identify animal species (Ahmed et al., 2018[1]; Knecht, 2012[22]). The here provided differential analysis of animal skin could also be used to identify animal species at criminal scene. This of course does not replace the species-specific PCR but it could be used as a supplementary method. Antemortem analysis of skin histology could also give a clue about the animal age (Bhattacharyya, 2017[7]; Brooks, 2016[9]). Age-induced reduction in the number of the sebaceous glands was previously reported in sheep (Warren et al., 1983[35]). Alteration of the arrangement of hair follicles and sebaceous glands, as well as the reduction of melanocyte number were also reported to be age-related changes in the hairless dog (Kimura and Doi, 1994[21]). Additional age-related changes in the skin of dogs and cats including alopecia, callus formation over pressure points, orthokeratotic hyperkeratosis of the epidermis, and atrophied hair follicles were previously reported (Scott et al., 2001[33]). From the surgical point of view, understanding the histological structure of the skin of each animal species may help to better evaluate the regeneration capacity and the prognosis of full-thickness skin wounds which results in extensive damage to the different skin layers and the underlying tissues. Based on the histological structure, clinical approaches to wound treatment could be better selected to promote rapid wound repair with functional and satisfactory scar tissue formation (Borena et al., 2015[8]).

Due to the rapid occurrence of postmortem changes in the skin (Reddy and Lowenstein, 2011[30]), its analysis could also provide valuable information regarding the time elapsed after death. This contrasts with the hair which resists the postmortem changes due to the high content of cysteine containing keratin (Harkey, 1993[17]). A limitation of the present study is that the samples were collected only from living animals and not after death. Nevertheless, our analysis provides a reference database of comparative skin histology. This will pave the way for follow up studies with postmortem specimens collected at various time points after death that will help to estimate the time elapsed after death.

In conclusion, the present study provides comprehensive information on the histological features of the skin of seven different animal species. The analysis revealed species-specific differential features that could be used in animal identification and other forensic investigations. 

## Declaration

### Acknowledgment 

The study was supported financially by the South Valley University, Qena, Egypt. 

### Conflict of interest 

The authors declare that they have no conflict of interest.

## Figures and Tables

**Table 1:**
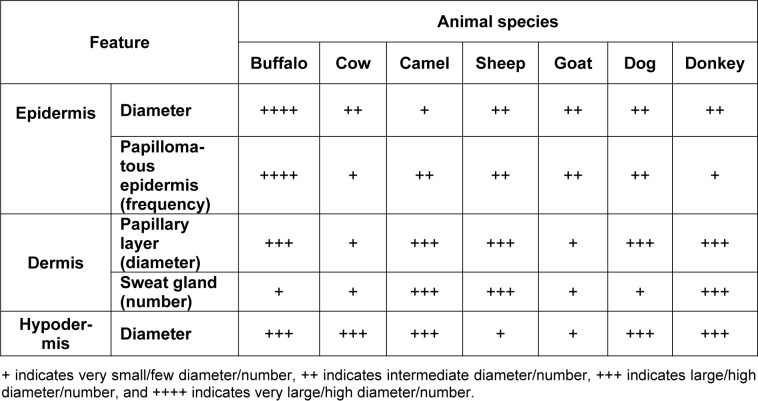
Differential histological features of the skin of buffalo, cow, camel, sheep, goat, dog, and donkey
